# Sub-micron microparticles with tunable fluorescence emission obtained via co-self-assembly of amidoximed polymeric ligands and lanthanide ions

**DOI:** 10.3389/fchem.2023.1209264

**Published:** 2023-05-17

**Authors:** Huandi Qiu, Qimeng Ran, Yun Bai, Wei He, Li Zheng, Cong Pan, Kun Jia, Yiguo Hu

**Affiliations:** ^1^ Department of Thyroid Surgery, State Key Laboratory of Biotherapy, West China Hospital, Sichuan University, Chengdu, China; ^2^ School of Materials and Energy, University of Electronic Science and Technology of China, Chengdu, China; ^3^ Institute for Radiation Protection, Taiyuan, Shanxi, China; ^4^ Biomedical Research Center, Chengdu eBond Pharmaceutical Technology Ltd., Chengdu, China; ^5^ Guizhou Kangqinchengping Biotechnology Company, Guiyang, Guizhou, China

**Keywords:** lanthanide ions, fluorescent microparticles, block copolymer, emulsion self-assembly, amidoxime, polymeric ligand

## Abstract

Lanthanide coordinating polymeric microparticles have witnessed increasing research interests during the past decades due to their versatile morphology and tunable fluorescent properties. Herein, we have synthesized an amidoximed block copolymer containing aromatic backbone and pendent amidoxime as well as carboxyl groups, which has been employed as the ligand to sensitize the intrinsic fluorescence emission of lanthanide ions of Tb^3+^ and Eu^3+^. Furthermore, the lanthanide coordinating polymeric microparticles showing tunable green and red emission fluorescence have been prepared via the emulsion confinement co-self-assembly of amidoximed polymeric ligands with Tb^3+^ and Eu^3+^. It is found that both the fluorescence emission and sizes of obtained fluorescent microparticles can be easily modulated in a wide range by tuning concentration of polymers and lanthanide ions, as well as emulsion evaporation temperature. Thanks to their tunable sizes (250–900 nm), fluorescence emission as well as presence of surface active functional groups, the present fluorescent microparticles would find potential applications in *in-vitro* detection, optical encoding and devices.

## 1 Introduction

Polymeric fluorescent microspheres are organic beads ranging from nanometers to micrometers in diameter and possessing tunable fluorescence emission properties (Yang et al., 2020). Thanks to their tunable and efficient luminescent emission as well as diverse morphological structure, fluorescent microspheres have great potential for applications in optical detection (He et al., 2023), labeling (Zhan et al., 2021), tracing (Kong et al., 2023), immobilization of enzymes (Ding et al., 2021), high-throughput drug screening (Sun et al., 2019) and immune medicine (Hooker et al., 2019). Therefore, in recent years, the research related to polymer fluorescent microspheres have received more and more attention from both the academic and industrial scenarios (Bian et al., 2019). Especially, the fluorophores used for preparation of polymeric fluorescent microspheres play the important role in determining their final performance.

According to the different fluorescent substances in the final beads, the polymeric fluorescent microspheres can be generally divided into inorganic fluorophore-polymeric microspheres and organic fluorophore-polymeric microspheres. Among them, the fluorescent microparticles obtained by using semiconductor quantum dots (QDs) (Jia et al., 2022b), organic dyes (You et al., 2019), rare earth metals (Pan et al., 2019), are the major types due to their specific advantage. For instance, QDs have stable resistance to photobleaching and narrow emission bands due to their quantum confinement effects (He et al., 2019). However, its drawbacks are cytotoxicity, induction of apoptosis and peroxidative stress, so its application in practical biomedical scenarios remains controversial (Masoomi et al., 2021). Organic dyes have diverse structure tailorability but normally suffer from low photostability and rapid photobleaching (Hoji et al., 2020). Interestingly, rare earth ions are widely used as luminescent materials due to their sharp emission bands and longer lifetime derived from the special electron layer structure of rare earth elements. Especially, lanthanides have unfilled 4f-5d electron states and thus possess abundant electron energy levels and long-lived fluorescence emission (Wang et al., 2021). Moreover, the fluorescence stability, selectivity and water solubility of lanthanide ions can be further enhanced by introducing appropriate polymeric ligands. Thus, the fluorescent lanthanide coordination polymeric micro/nano-structures can be obtained using direct coordination of lanthanide ions with the functional repeating side groups of the polymer (Ran et al., 2023).

Besides of fluorescence emission control, the size modulation of polymeric microparticles is another important aspect for their application. When compared to the widely used solvent exchange induced nano-precipitation protocol, the restricted self-assembly of block copolymer in a three dimensionally confined emulsion drop allows preparation of polymeric microparticles with more diverse surface morphology and tunable sizes (Xu et al., 2015; Shin et al., 2020). For instance, a range of polymeric microparticles with Janus, porous, football-, lens-, and onion-like morphologies have been prepared via the emulsion confinement self-assembly of classical block copolymer of PS-b-P4VP (Deng et al., 2016; Cui et al., 2019; Ku et al., 2019; Lee et al., 2019; Steinhaus et al., 2019). More interestingly, some recently published works have proven that co-self-assembly of block copolymer with inorganic entities (e.g., plasmonic nanoparticles, magnetic nanoparticles and quantum dots), not only rendered the resultant polymeric microparticles with additional optical or magnetic functionalities, but also enhanced the overall structural stability of the resultant hybrid microparticles (He et al., 2018; Yan et al., 2019; He et al., 2021). However, it is found that the research work on combination of lanthanide ions coordination with block copolymer during emulsion confinement self-assembly is still quite limited. One of possible reasons would be ascribed to the challenging of designing a specific block copolymer that exhibit both robust self-assembly tendency and lanthanide ions sensitization capacity.

In this work, we designed and synthesized a block co-polyarylene ether sulfone derivative bearing pendent nitrile and carboxyl groups (abbreviated as PENS hereafter). Next, the synthesized PENS was further transformed into amidoximed block copolymer (named as AO-PENS) to bring more reaction sites on the macromolecular chains. To further enrich the fluorescent properties of the polymeric microparticles, lanthanide rare earth metal ions (Tb^3+^/Eu^3+^) were introduced during the emulsion confinement self-assembly of AO-PENS. Thanks to the coordination between pendent groups of AO-PENS with lanthanide ions, the fluorescent polymer microspheres (Ln-AO-PENS) were prepared and their surface morphology, size evolution and fluorescence properties can be readily modulated by changing different parameters during self-assembly.

## 2 Materials and methods

### 2.1 Materials

N-methylpyrrolidine (NMP), N,N-dimethylformamide (DMF), hydrochloric acid (HCl), toluene, bisphenol A (BPA), sodium hydroxide (NaOH), sodium dodecylbenzene sulfonate (SDS), cetyltrimethylammonium bromide (CTAB) and dichloromethane (DCM) were purchased from Chron Chem. 2,6-difluorobenzonitrile (DFBN) and terbium chloride hexahydrate (TbCl_3_·6H_2_O) were acquired from Shanghai Aladdin. Europium chloride hexahydrate (EuCl_3_·6H_2_O) was acquired from Shanghai Xushuo. 4,4′-dichlorodiphenyl sulfone (DCDPS) was obtained from Chengdu Haihong. Potassium carbonate (K_2_CO_3_), phenolphthalein (PP) and zinc powder were purchased from Tianjin Bodi. Phenolphthalin (PPL) was prepared by the synthesis of phenolphthalein, zinc powder and sodium hydroxide.

### 2.2 Synthesis of polyarylene ether nitrile sulfone (PENS)

Polyarylene ether nitrile sulfone multiblock copolymers (PENS) were synthesized via the nucleophilic substitution polycondensation reaction (Li et al., 2022), where hydrophobic oligomeric chain segment A and hydrophilic oligomeric chain segment B were synthesized first. In a typical synthesis, BPA-based poly (aryl ether nitrile) was used as hydrophobic oligomer A and PPL-based poly (sulfone) was used as hydrophilic oligomer B for block copolymerization. Hydrophobic segment A is synthesized from BPA and DFBN. In particular, BPA (4.7028 g, 0.0206 mol), DFBN (2.782 g, 0.02 mol) and K_2_CO_3_ (4.2704 g, 0.0309 mol) were added to a three-necked flask and 16 mL of NMP with 8 mL of toluene was added as the solvent for the reaction system, followed by continuous stirring and slow heating of the reaction mixture to 145°C for 3 h. Then, the reaction mixture was further heated at 180°C for 1 h to obtain hydrophobic oligomeric chain segment A. Likewise, hydrophilic oligomer chain segment B was obtained by adding PPL (6.4068 g, 0.02 mol), DCDPS (5.8868 g, 0.0205 mol), K_2_CO_3_ (6.219 g, 0.045 mol) to a flask containing NMP (18 mL) and toluene (8 mL), heating to 140°C for 3 h and then heating to 180°C for a further reaction of 1 h. After the temperature of the oligomer in the two flasks had dropped to around 100°C, oligomer B and the solvent were transferred to the reaction vessel of oligomer A with continuous stirring. Next, the temperature of mixture was slowly raised to 180°C at a rate of 10°C/h to maintain the reaction for 3 h. The reaction products were then transferred to a beaker containing dilute hydrochloric acid to obtain the crude PENS product. The crude PENS product was crushed into powder, condensed and refluxed three times using ethanol and pure water respectively, and finally dried overnight in a vacuum oven at 80 °C to obtain PENS powder.

### 2.3 Preparation of amidoximed PENS (AO-PENS)

To obtain the amidoximed polyarylene ether nitrile sulfone (AO-PENS) (Ran et al., 2023), hydroxylamine hydrochloride (15 g), PNES (10 g) and DMF (50 mL) were added to a flask, then NaHCO_3_ was added to adjust the pH of the system to neutral and the reaction was carried out in an oil bath at 120°C for 15 h. The reaction product was washed in dilute hydrochloric acid and dried in an oven at 80°C for 12 h to obtain AO-PENS.

### 2.4 Fabrication of AO-PENS microspheres

First, TbCl_3_·6H_2_O or EuCl_3_·6H_2_O (100 μL) at a certain concentration was added to a brown bottle containing 10 mL of CTAB aqueous solution (continuous phase) with a concentration of 3 mg/mL and allowed to dissolve completely. After complete dissolution, AO-PENS solid powder (5 mg) was dissolved in DMF (100 μL) and added to DCM (900 μL) to form the oil phase, which was then injected into the continuous phase at room temperature and stirred continuously at a magnetic stirring rate of 1,000 rpm/min for 6 h to fully emulsify the polymer. The sample bottle was then opened to allow the DCM in the system to evaporate completely to obtain the assembled product. Finally, the assembled products were centrifuged at high speed and washed three times with deionized water to obtain lanthanide-coordinated amidoxime polyarylene ether nitrile sulfone nanospheres (Ln-AO-PENS).

### 2.5 Characterization

NMR spectroscopy (^1^H NMR, AVANCE III 400M, Burke, Germany) and Fourier infrared spectroscopy (FTIR, Nicolet Is10, Thermo Fisher, US) were used to analyze the chemical structure of the synthesized PENS, respectively. The molecular weights of PENS and AO-PENS were characterized by gel permeation liquid chromatography (GPC, Waters 1515/2414, Waters, US). The thermal property of PENS and AO-PENS were characterized by differential scanning calorimeter (DSC-Q100, TA, US) and thermogravimetric analyzer (TGA-Q50, TA, US), respectively. SEM images of Ln-AO-PENS microspheres were obtained using a JEOL scanning electron microscope (SEM, JSM-6490LV, Japan). The photographs of Ln-AO-PENS microspheres in the cuvette were obtained at the excitation wavelength of 302 nm in dark box. Fluorescence spectrophotometer (SPF, Hitachi, F-4600, Japan) was used to test the fluorescence spectrum of the samples, while the fluorescent yields and lifetime of Ln-AO-PENS microparticles were determined by using a PicoQuant FT-300 and an Edinburgh Instruments FLS980 instrument, respectively. The electronic structure of the samples was characterized by X-ray photoelectron spectrometry (XPS, Escalab Xi+, Thermo Fisher, Germany).

## 3 Results and discussion

**SCHEME 1:**
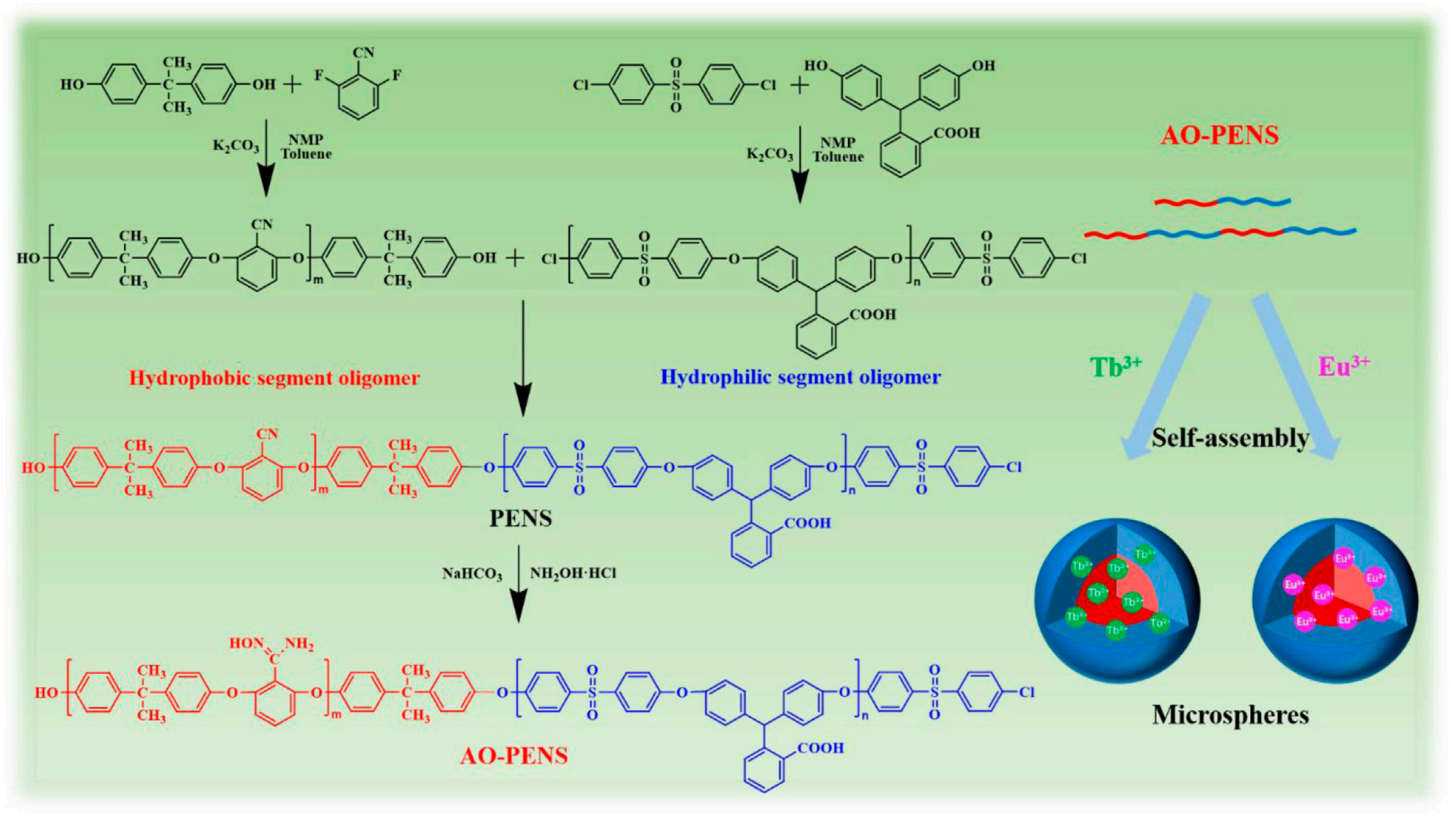
The synthetic route of AO-PENS block copolymer and the schematic diagram for preparation of Ln-AO-PENS fluorescent microspheres.

As is well known, amphiphilic block copolymers can be self-assembled into polymeric microspheres of different sizes and morphology via the emulsion solvent evaporation method. On the other hand, rare earth metal elements have intense, long-lived and narrow absorption bands of fluorescence emission due to their unique electronic configurations and strong spin-orbit coupling (Jia et al., 2022a). Among them, lanthanide elements of terbium (Tb) and europium (Eu) can emit green and red luminescence after being sensitized with appropriate organic/polymeric ligand. Thus, when the polymeric ligands with intrinsic blue-emitting is employed to sensitize the intrinsic fluorescence of Tb and Eu, the obtained polymeric microparticles should exhibit diverse fluorescent emission colors. For this reason, the amidoximed polyarylene ether nitrile sulfone (AO-PENS) contains carboxylic acid groups and amidoxime groups was synthesize and coordinated with Tb and Eu ions during the emulsion evaporation induced self-assembly process. Specifically, when rare earth ions of Tb^3+^ and Eu^3+^ are introduced into the AO-PENS block copolymer in confined emulsion droplet (Scheme 1), the expected *in situ* cross-linking reaction between carboxylic acid groups and rare earth ions during microsphere formation can not only further improve the structural stability of fluorescent polymer microspheres, but also endow final polymeric microspheres with long-lived fluorescent emission (with a fluorescent yield and lifetime of 13.1%, 1,263 μs for Tb-AO-PENS and 14.1%, 878 μs for Eu-AO-PENS, respectively).

**FIGURE 1:**
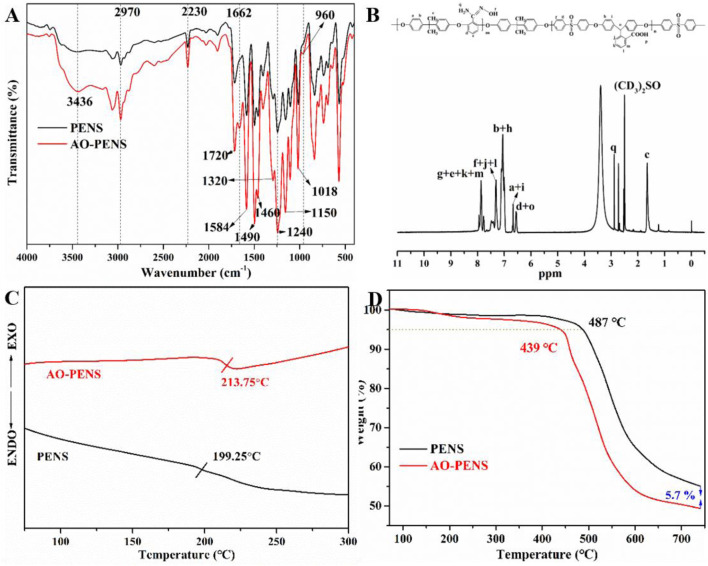
The chemical structures and thermal properties of PENS and AO-PENS. FTIR spectra **(A)**, DSC curves **(C)**, TGA curves **(D)** of PENS and AO-PENS, ^1^H NMR spectrum **(B)** of AO-PENS.

First, the structures of PENS and AO-PENS were characterized by FTIR, ^1^H NMR, DSC and TGA, as shown in Figure 1. From the FTIR spectra of PENS and AO-PENS in Figure 1A, it is clear that the absorption peak of the C=N bond appears at 1,662 cm^−1^, the peak of the N-O bond appears at 960 cm^−1^, and the stretching vibration absorption peak of the amino group (-NH_2_) appears at 3,436 cm^−1^ (Pan et al., 2022). The absorption at 2,970 cm^−1^ is the stretching vibration characteristic peak of methyl (C-H) in the PENS and AO-PENS structures, and the stretching vibration characteristic peak at 2,230 cm^−1^ is ascribed to the nitrile group (-CN). The skeletal vibrational characteristic absorption peaks attributed to benzene ring were observed around 1,584 cm^−1^, 1,490 cm^−1^ and 1,460 cm^−1^, and the absorption peaks corresponding to sulfone group (-S=O) are detected at 1,320 cm^−1^ and 1,150 cm^−1^. The peak at 1720 cm^−1^ is attributed to the stretching vibration of carbonyl group (-C=O) in carboxyl group, and the peaks at 1,240 cm^−1^ and 1,018 cm^−1^ are attributed to the asymmetric and symmetric stretching vibration absorption of Ar-O-Ar. The ^1^H NMR hydrogen spectrum of AO-PENS (Figure 1B) shows a chemical shift near 2.88 ppm, which belongs to the ammonia base on the characteristic peak of hydrogen atoms (Liu et al., 2020). It is clear from Figure 1C that the glass transition temperature of AO-PENS is 213.75°C, which is 14°C higher compared to that of PENS, indicating that the heat resistance of both PENS and AO-PENS is excellent, and the molecular chains of AO-PENS are more tightly entangled and may have undergone some crosslinking. It can be observed from the TGA curves (Figure 1D) that both PENS and AO-PENS copolymers have excellent thermal stability with their 5% thermal decomposition temperatures exceeding 430°C. The decrease of thermal decomposition temperature of AO-PENS may be due to the partial conversion of pendent -C≡N to -C=N, which declines the inter-molecular entanglement. In short, AO-PENS copolymer has been successfully synthesized.

**FIGURE 2:**
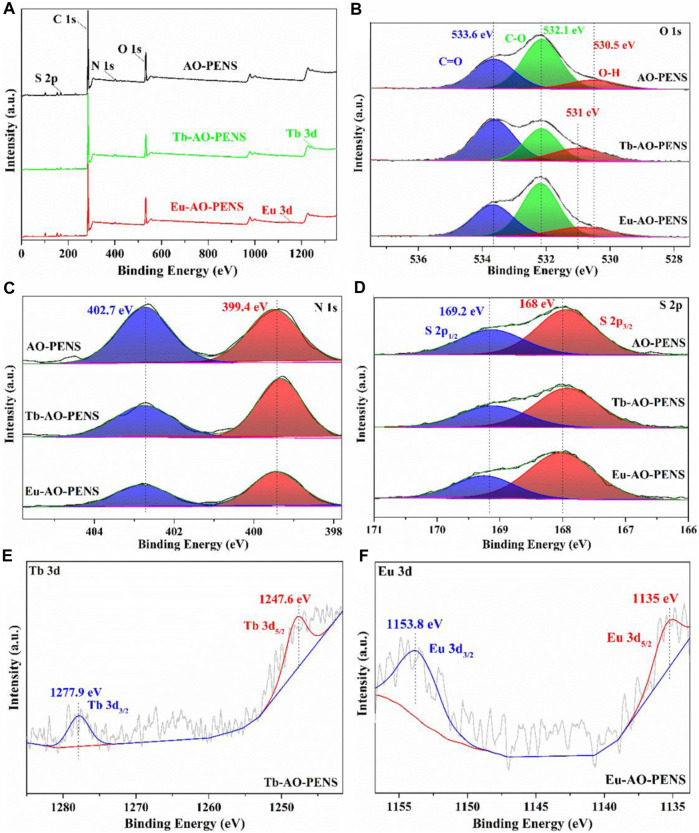
X-ray photoelectron spectra of AO-PENS and Ln-AO-PENS microspheres. **(A)** Full scale XPS spectra of AO- PENS and Ln-AO-PENS microspheres, high resolution spectrum of **(B)** O 1s, **(C)** N 1s, **(D)** S 2p, **(E)** Tb 3d, **(F)** Eu 3d.

Next, the prepared Ln-AO-PENS fluorescent microspheres were characterized by XPS spectroscopy. As seen in the full scale XPS spectrum (Figure 2A), all chemical elements are detected from AO-PENS and Ln-AO-PENS microspheres. More importantly, a peak shift belonging to the O-H group is observed in the high resolution O 1s spectrum (Figure 2B), specifically from 530.5 eV for AO-PENS to 531 eV for Ln-PENS. But the N 1s peaks (399.4 eV and 402.7 eV) and S 2p peaks (169.2 eV for S 2p_3/2_ and 168 eV for S 2p_3/2_) of AO-PENS and Ln-AO-PENS microspheres are not shifted, as shown in Figure 2C; Figure 2D. Meanwhile, the XPS spectra of Tb and Eu are shown in Figure 2E; Figure 2F. The 3d orbitals of Tb split into Tb 3d_3/2_ and Tb 3d_5/2_ with binding energies of 1,247.6 eV and 1,277.9 eV (Ca et al., 2021), respectively, while the 3d orbitals of Eu split into Eu 3d_3/2_ and Eu 3d_5/2_ with binding energies of 1,135 eV and 1,153.8 eV (Hasabeldaim et al., 2020), respectively. The above data confirm that the lanthanide ions are coordinated with AO-PENS ligands to prepare the Ln-AO-PENS fluorescent microspheres.

### 3.1 Effect of different Tb^3+^/Eu^3+^ ion concentrations on fluorescent microspheres

**FIGURE 3:**
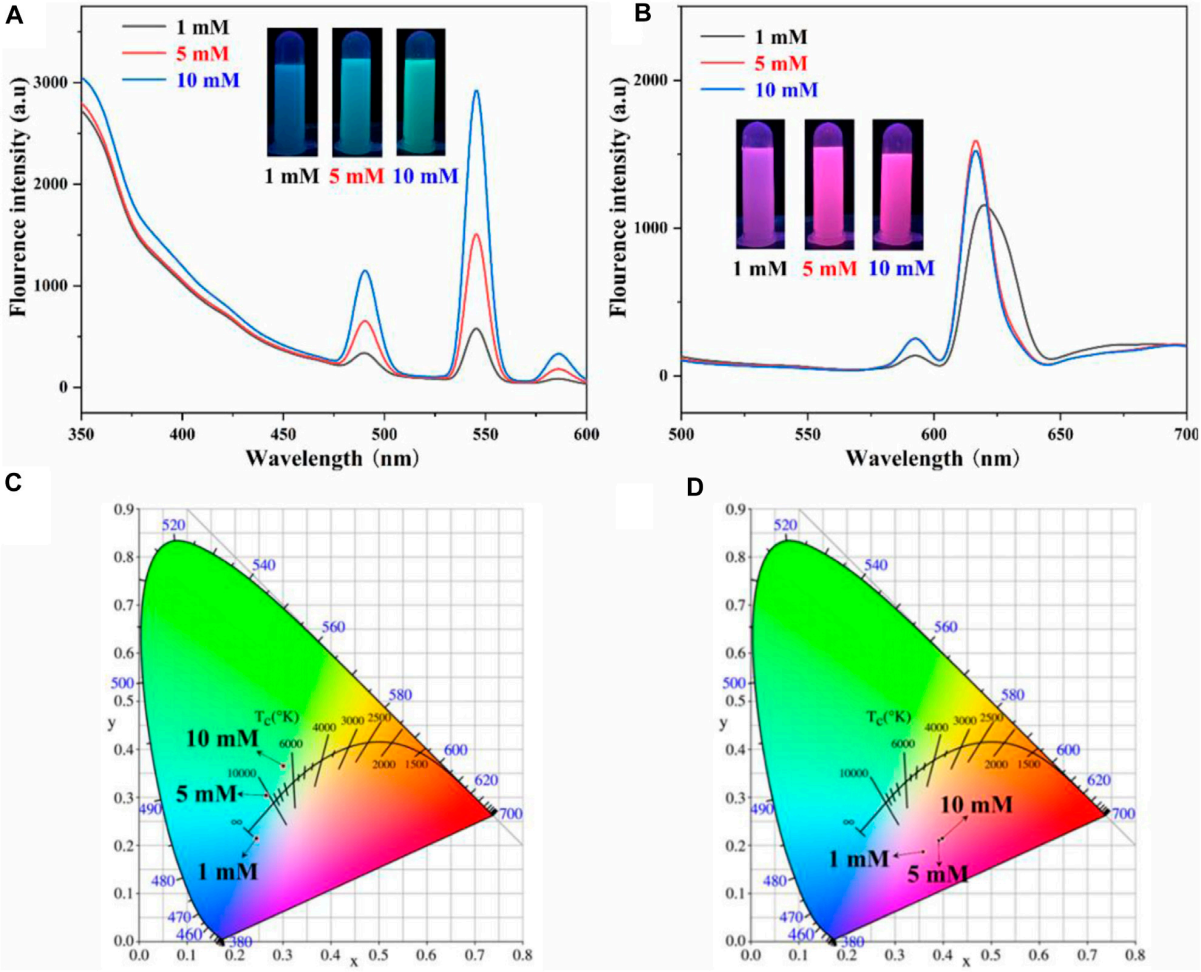
Fluorescence emission spectra and CIE 1931 chromaticity coordinates of Tb-AO-PENS **(A,C)** and Eu-AO-PENS **(B,D)** microparticles prepared at different Tb^3+^/Eu^3+^ ion concentrations.

Since the AO-PENS block copolymer contains amidoxime groups, which have been reported to have coordination with rare earth ions. Therefore, the effects of different rare earth ion concentrations on the fluorescence properties of lanthanide AO-PENS (Ln-AO-PENS) microspheres were investigated, as shown in Figures 3A,B. Similarly, the fluorescence intensity of Ln-AO-PENS fluorescent microspheres also increased with the increase of rare earth ion concentration. This is also verified by the CIE color coordinates plots shown in Figure 3C, the color coordinates of Tb^3+^-coordinated AO-PENS (Tb-AO-PENS) microspheres for 1 mM, 5 mM, and 10 mM Tb^3+^ conditions are calculated as (0.2453, 0.2151), (0.2658, 0.3036) and (0.3007, 0.3651). As in Figure 3D, the Eu^3+^-coordinated AO-PENS (Eu-AO-PENS) microsphere color coordinates for 1 mM, 5 mM and 10 mM Eu^3+^ conditions are determined as (0.3591, 0.187), (0.3923, 0.2107) and (0.39930, 2,147). Based on the above experimental results, it is clear that an increase in the concentration of Tb^3+^/Eu^3+^ ions leads to an enhancement of the fluorescence intensity and easy modulation of emitting colors of Ln-AO-PENS microspheres.

**FIGURE 4:**
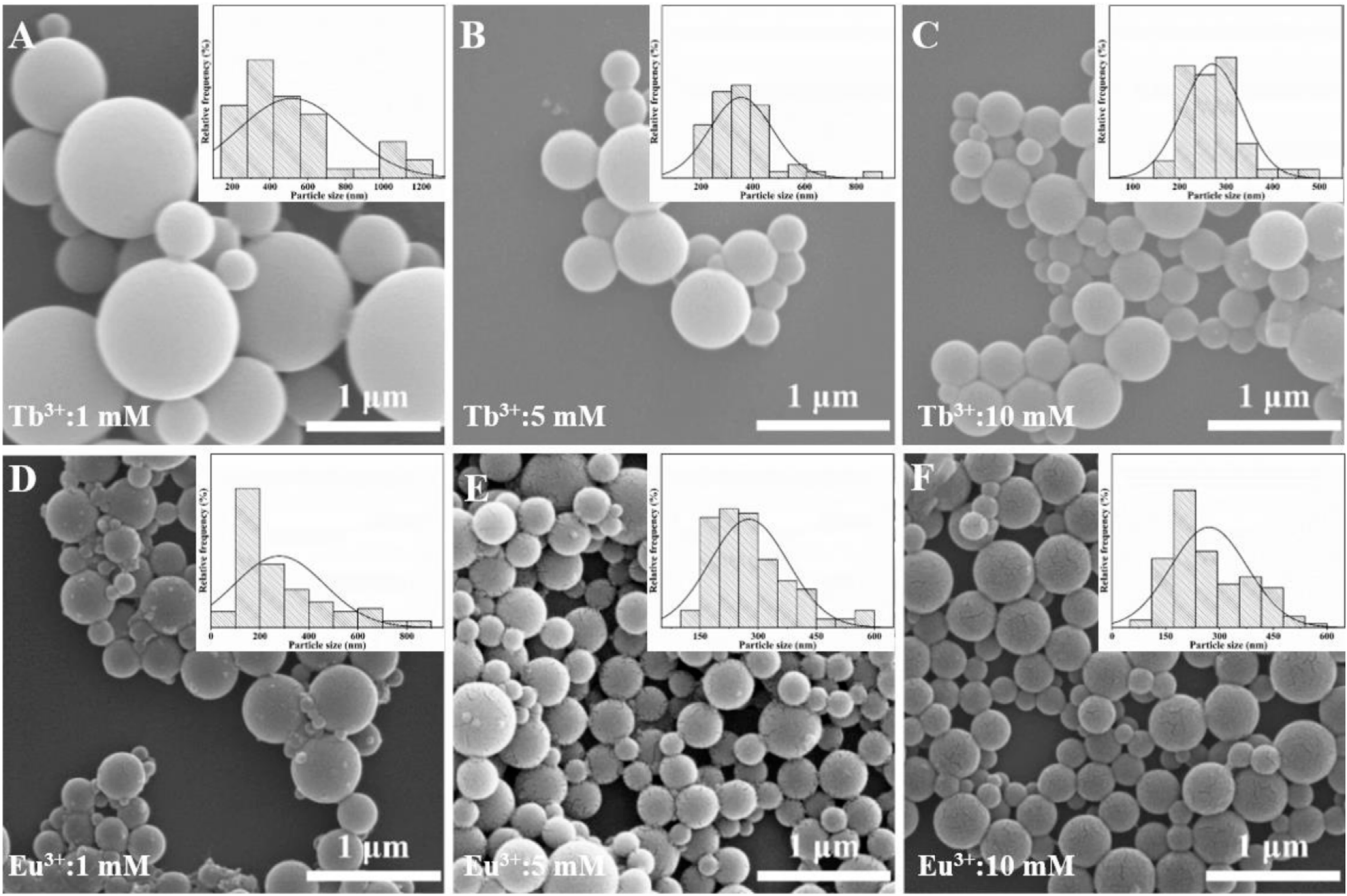
SEM images of Ln-AO-PENS microparticles prepared using different concentrations of lanthanide ions of **(A)** 1 mM Tb^3+^, **(B)** 5 mM Tb^3+^, **(C)** 10 mM Tb^3+^, **(D)** 1 mM Eu^3+^, **(E)** 5 mM Eu^3+^ and **(F)** 10 mM Eu^3+^.

The effect of Tb^3+^/Eu^3+^ concentration on the morphology of AO-PENS fluorescent microspheres was investigated by SEM and the results were shown in Figure 4. From Figures 4A–C, it can be seen that the size of AO-PENS microspheres decreases slightly with the increase of Tb^3+^ concentration, and this trend can also be found in the particle size distribution of Tb-AO-PENS microspheres in the corresponding inset images of Figure 4. More specifically, the average size of microspheres decreases from 600 nm to 300 nm when the Tb^3+^ concentration increases from 1 mM to 5 mM. The size evolution of Eu-AO-PENS microspheres is highly dependent on the concentration of Eu^3+^ according to the SEM and histograms of particle size distribution in Figures 4D–F. More specifically, when the concentration of Eu^3+^ is 1 mM, the resulting microspheres have a rough surface and a wide range of size distribution (from 100 nm up to 900 nm), while as the increasing of Eu^3+^ concentration to 10 mM, the size distribution of resultant polymeric microspheres is decreased to 150–450 nm. The possible reason for this evolution would be rationalized as follow. In the one hand, when low concentration of lanthanide ions is used, the excess amount of AO-PENS can also self-assembled into pristine polymeric beads, which actually leads to the inhomogeneous population into resultant microparticles. On the other hand, the lanthanide ions hydrolysis will lead to protonation of surfactant (CTAB), which in turn contribute to smaller emulsion droplet with good stability and eventually to smaller microparticles.

### 3.2 Effect of different polymer concentrations on fluorescent microspheres

**FIGURE 5:**
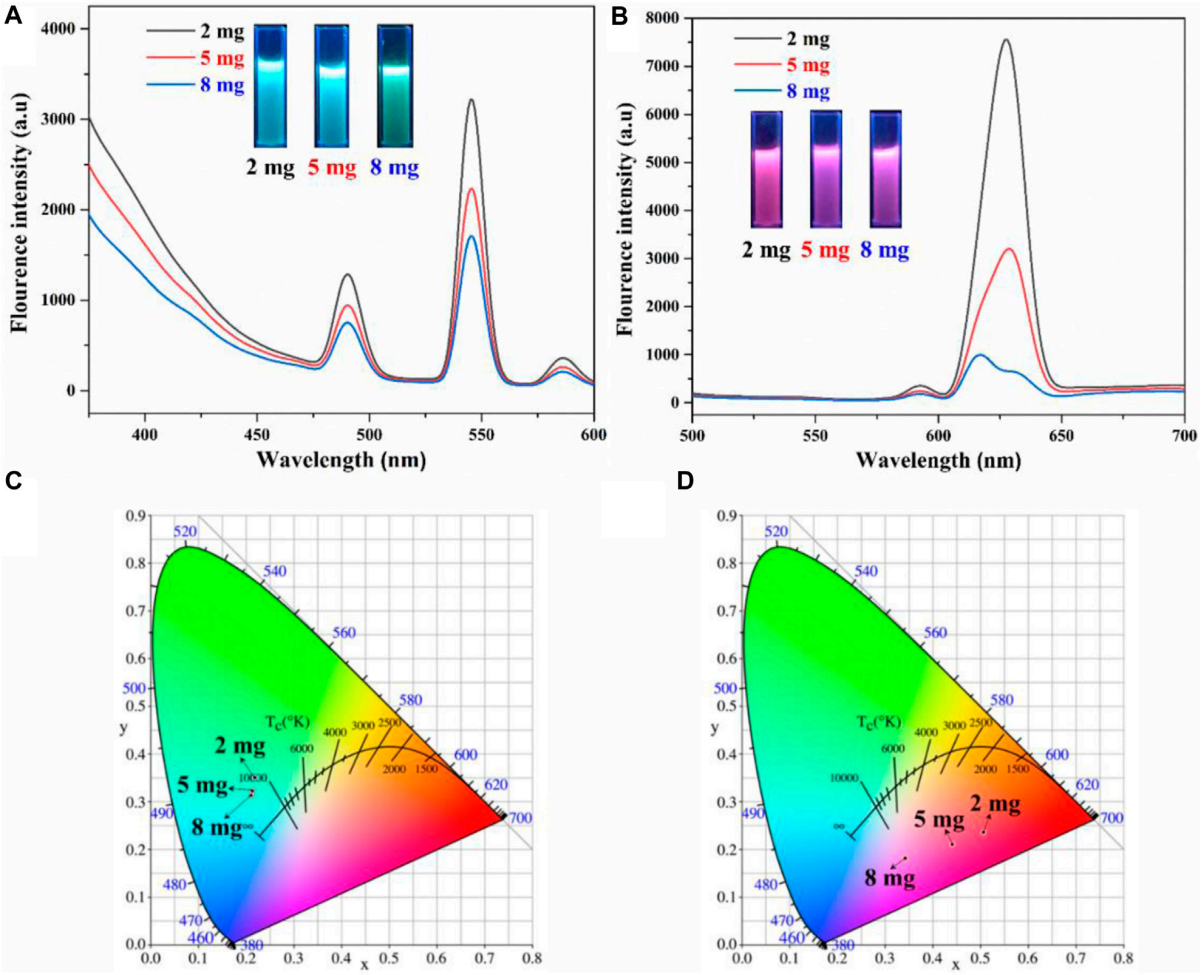
Fluorescence emission spectra and CIE 1931 chromaticity coordinates of Tb-AO-PENS **(A,C)** and Eu-AO-PENS **(B,D)** particles prepared at different AO-PENS concentrations.

Besides of lanthanide ions concentrations, the effect of different AO-PENS content conditions on the fluorescence properties and morphology of Ln-AO-PENS microspheres were also conducted. According to the fluorescent properties shown in Figure 5, the fluorescence intensity of Ln-AO-PENS fluorescent microspheres is increased with the decrease of AO-PENS content, and the CIE color coordinates plot showed a clear pattern: the lower the content of AO-PENS, the resultant Tb-AO-PENS/Eu-AO-PENS microspheres exhibit greener/redder the fluorescence emission color. The Tb-AO-PENS microsphere obtained by using 2 mg, 5 mg and 8 mg of AO-PENS had fluorescent color coordinates of (0.3619,0.4137), (0.2746,0.3835) and (0.2638,0.3187), while the fluorescent microsphere color coordinates of Eu-AO-PENS for 2 mg, 5 mg and 8 mg AO-PENS were (0.55,0.2624), (0.4205,0.2207) and (0.339,0.1912), respectively.

**FIGURE 6:**
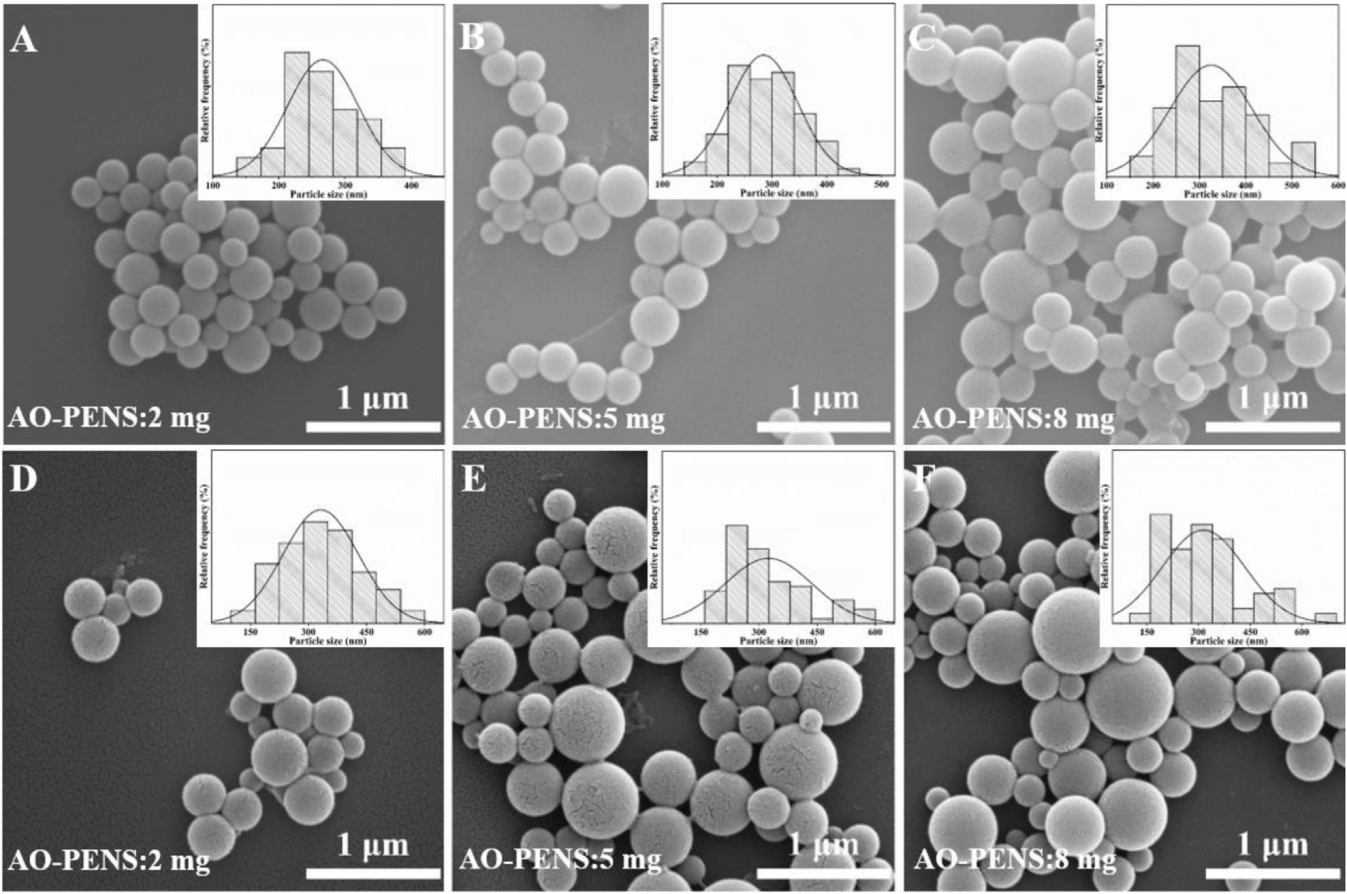
SEM image of Tb-AO-PENS microparticles **(A,B,C)** and Eu-AO-PENS microparticles **(D,E,F)** prepared with 2 mg AO-PENS **(A,D)**, 8 mg AO-PENS **(B,E)** and 10 mg AO-PENS **(C,F)**, respectively.

The effect of different AO-PENS contents on the morphology of the fluorescent microspheres was investigated and the microscopic morphology of the microspheres obtained was shown in Figure 6. The average size of Tb-AO-PENS microspheres increases from 250 nm to approximately 375 nm when the AO-PENS content is increased from 2 mg to 8 mg, and the average size of Eu-AO-PENS microspheres increased from 300 nm to approximately 400 nm. In short, as the increasing amount of polymer is involved during the self-assembly, the larger hybrid polymeric microparticles with declined fluorescence emission are obtained.

### 3.3 Effect of different volatilization rates on fluorescent microspheres

**FIGURE 7:**
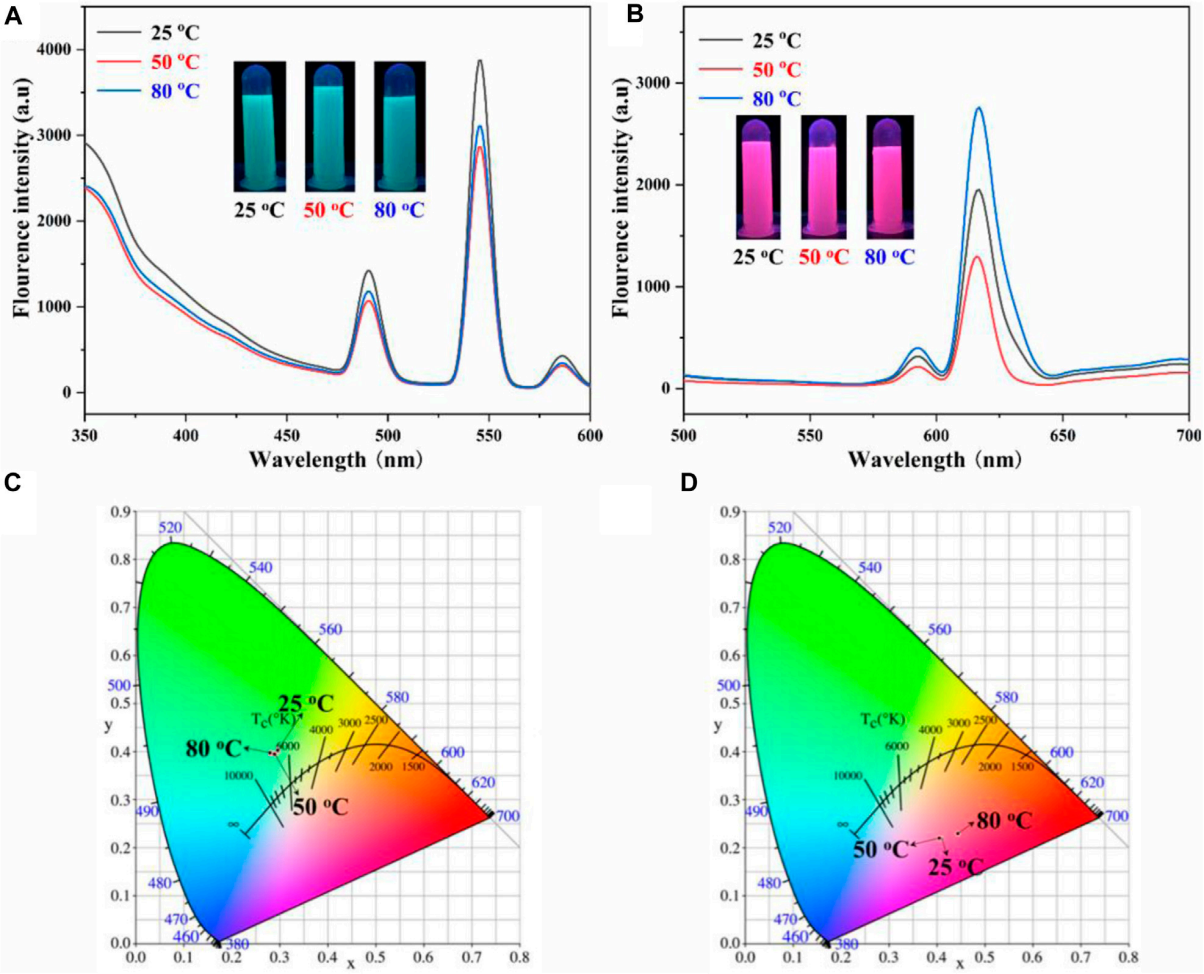
Fluorescence emission spectra and CIE 1931 chromaticity coordinates of Tb-AO-PENS **(A,C)** and Eu-AO-PENS **(B,D)** particles at different evaporation tempartures.

In addition, the effect of organic solvents evaporation temperature on the fluorescence properties of Ln-AO-PENS microspheres was investigated by recording their fluorescence emission spectra. It can be seen from Figure 7A that the fluorescence intensity of Tb-AO-PENS microspheres is the highest when the evaporation temperature is 25°C. However, the fluorescence intensity of Eu-AO-PENS microspheres decreases and then increases with the increase of temperature, and an evaporation temperature of 80°C allows preparation of polymeric microparticles with strongest emission (Figure 7B). CIE color coordinates of polymeric microparticles prepared at different temperatures were also obtained according to the recorded fluorescence spectral data. As shown in Figures 7C,D, the fluorescence microsphere color coordinates of Tb-AO-PENS and Eu-AO-PENS hybrid microparticles, prepared using an evaporation temperature 25°C, 50°C, and 80°C, were calculated as (0.2957, 0.4025), (0.2888, 0.3947), (0.2802, 0.3967), and (0.4097, 0.2181), (0.4052, 0.2204), (0.4441, 0.2296), respectively.

**FIGURE 8:**
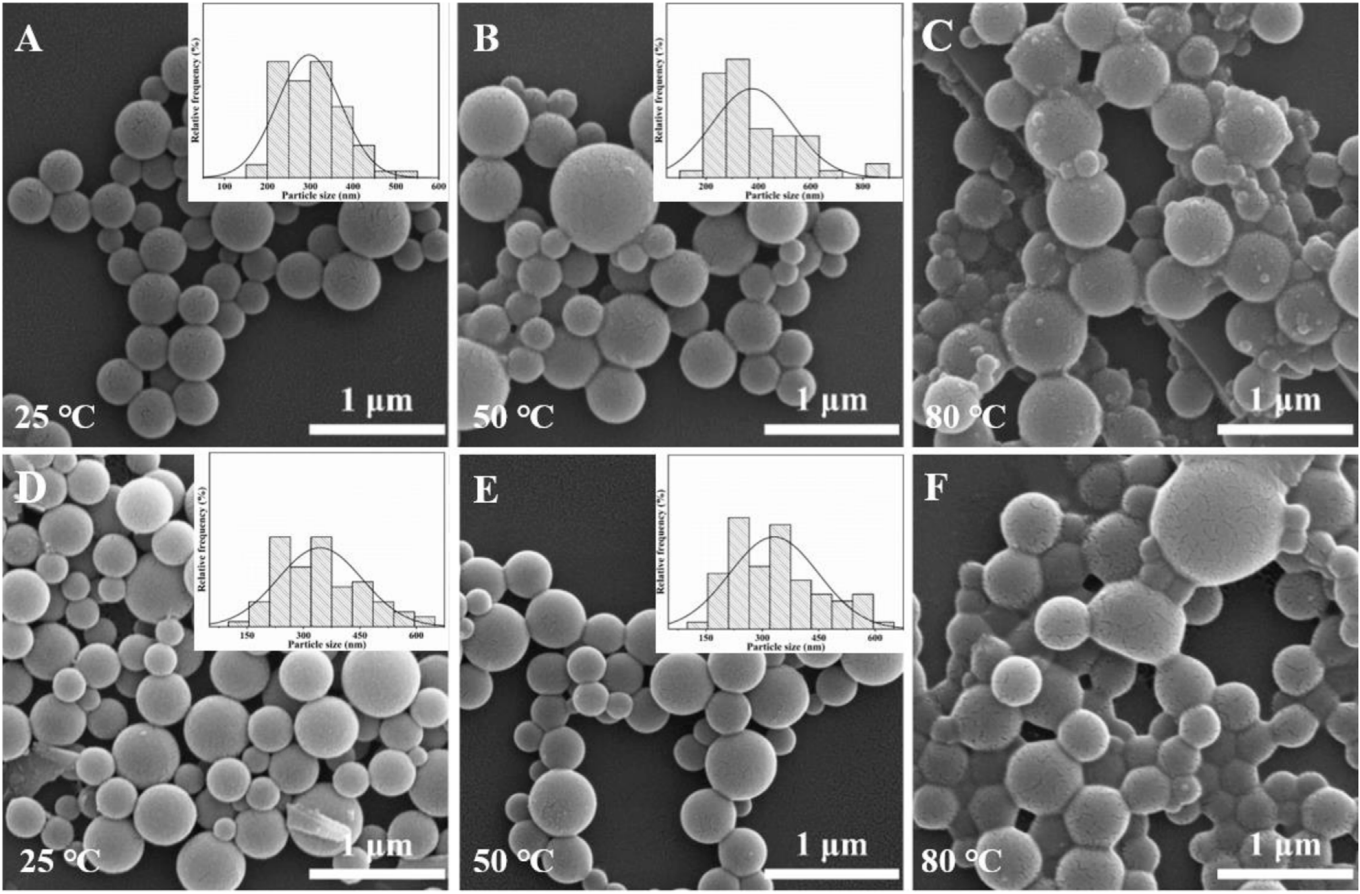
SEM images of Tb-AO-PENS microparticles **(A,B,C)** and Eu-AO-PENS microparticles **(D,E,F)** prepared at evaporation temperatures of 25°C **(A,D)**, 50°C **(B,E)** and 80°C **(C,F)**, respectively.

Finally, the effects of evaporation temperature on the surface morphology of the Ln-AO-PENS fluorescent microspheres were investigated using a fixed AO-PENS content of 5 mg and a Tb^3+^/Eu^3+^ concentration of 10 mM. It can be seen from Figure 8 that as the increasing of evaporation temperature, the surface of Ln-AO-PENS microspheres becomes rougher, and more obvious coalescence among polymeric microparticles are detected. Although the specific reason for this still requires additional experiments, we assume that when the self-assembly is conducted at a higher evaporation temperature, the faster evaporation of organic solvent would result to stronger alternation of the surfactant stabilized emulsion droplets, finally leading to the fusion of some microspheres to form large-sized microspheres. In addition, the increasing fusion of emulsion droplets at even higher evaporation temperature would eventually lead to the enhanced adhesion or deformation between the microspheres. Actually, according to the particle size distribution analyses shown in Figure 8, it is clear that the average size of microspheres is basically independent of solvent evaporation temperature.

## 4 Conclusion

In this work, a reactive block copolymer containing aromatic backbone and lanthanide ions coordinating carboxyl as well as amidoxime groups in side chain was synthesized via nucleophilic substitution polycondensation and subsequent post-modification. The obtained copolymer of AO-PENS was further used as the ligand to prepare Ln-AO-PENS fluorescent microparticles by the emulsion solvent evaporation induced self-assembly of AO-PENS in the presence of Tb^3+^/Eu^3+^. Then the chemical structures of prepared fluorescent microparticles were characterized and verified. Moreover, we found that the fluorescence properties and microscopic morphology of the resultant microspheres can be readily controlled by changing the concentration of lanthanide ions and polymer as well as evaporation temperature during the self-assembly process. Thanks to the tunable size, fluorescent emission spectra/color as well as surface reactive functional groups, the obtained polymeric microspheres would find potential applications in the fields of optical diagnostics, multiple detection and anti-counterfeiting, which are currently in progress in our lab.

## Data Availability

The original contributions presented in the study are included in the article/Supplementary Material, further inquiries can be directed to the corresponding authors.

## References

[B1] Bian Y. Wei Z. Wang Z. Tu Z. Zheng L. Wang W. (2019). Development of biodegradable polyesters based on a hydroxylated coumarin initiator towards fluorescent visible paclitaxel-loaded microspheres. J. Mater Chem. B 7, 2261–2276. 10.1039/c8tb02952k 32254675

[B2] Ca N. X. Vinh N. D. Bharti S. Tan P. M. Hien N. T. Hoa V. X. (2021). Optical properties of Ce^3+^ and Tb^3+^ co-doped ZnS quantum dots. J. Alloys Compd. 883, 160764. 10.1016/j.jallcom.2021.160764

[B3] Cui T. Li X. Dong B. Li X. Guo M. Wu L. (2019). Janus onions of block copolymers via confined self-assembly. Polymer 174, 70–76. 10.1016/j.polymer.2019.04.062

[B4] Deng R. Li H. Zhu J. Li B. Liang F. Jia F. (2016). Janus nanoparticles of block copolymers by emulsion solvent evaporation induced assembly. Macromolecules 49, 6409–6429. 10.1021/acs.macromol.5b02507

[B5] Ding G. Wang A. Shi X. Li J. You L. Wang S. (2021). Preparation of multiple-spectra encoded polyphosphazene microspheres and application for antibody detection. Polym. Bull. 79, 6409–6429. 10.1007/s00289-021-03811-w

[B6] Hasabeldaim E. H. H. Ntwaeaborwa O. M. Kroon R. E. Coetsee-Hugo E. Swart H. C. (2020). Pulsed laser deposition of a ZnO:Eu^3+^ thin film: Study of the luminescence and surface state under electron beam irradiation. Appl. Surf. Sci. 502, 144281. 10.1016/j.apsusc.2019.144281

[B7] He J. Li Z. Zhao R. Lu Y. Shi L. Liu J. (2019). Aqueous synthesis of amphiphilic graphene quantum dots and their application as surfactants for preparing of fluorescent polymer microspheres. Colloids Surf. A Physicochem Eng. Asp. 563, 77–83. 10.1016/j.colsurfa.2018.11.064

[B8] He J. Yu L. Jiang Y. Lu L. Han Z. Zhao X. (2023). Encoding CsPbX_3_ perovskite quantum dots with different colors in molecularly imprinted polymers as fluorescent probes for the quantitative detection of Sudan I in food matrices. Food Chem. 402, 134499. 10.1016/j.foodchem.2022.134499 36303389

[B9] He X. Jia K. Marks R. Hu Y. Liu X. (2021). 3D confined self-assembling of QD within super-engineering block copolymers as biocompatible superparticles enabling stimulus responsive solid state fluorescence. Nano Res. 14, 285–294. 10.1007/s12274-020-3086-0

[B10] He X. Shen X. Li D. Liu Y. Jia K. Liu X. (2018). Dual-mode fluorescence and magnetic resonance imaging nanoprobe based on aromatic amphiphilic copolymer encapsulated CdSe@CdS and Fe_3_O_4_ . ACS Appl. Bio Mater 1, 520–528. 10.1021/acsabm.8b00240 35016388

[B11] Hoji A. Muhammad T. Wubulikasimu M. Imerhasan M. Li H. Aimaiti Z. (2020). Syntheses of BODIPY-incorporated polymer nanoparticles with strong fluorescence and water compatibility. Eur. Polym. J. 141, 110058. 10.1016/j.eurpolymj.2020.110058

[B12] Hooker J. P. Delafresnaye L. Barner L. Barner-Kowollik C. (2019). With polymer photoclicks to fluorescent microspheres. Mater Horizons 6, 356–363. 10.1039/c8mh01078a

[B13] Jia K. Ji Y. He X. Xie J. Wang P. Liu X. (2022a). One-step fabrication of dual functional Tb^3+^ coordinated polymeric micro/nano-structures for Cr(VI) adsorption and detection. J. Hazard Mater 423, 127166. 10.1016/j.jhazmat.2021.127166 34560484

[B14] Jia K. Yi K. Zhang W. Yan P. Zhang S. Liu X. (2022b). Carbon nanodots calibrated fluorescent probe of QD@amphiphilic polyurethane for ratiometric detection of Hg (II). Sens. Actuators B Chem. 370, 132443. 10.1016/j.snb.2022.132443

[B15] Kong W. Feng H. Qian X. Chen Y. Deng M. Zhang P. (2023). Facile and scalable generation of fluorescent microspheres using a microfluidic electrojetting device. Sens. Actuators B Chem. 378, 133106. 10.1016/j.snb.2022.133106

[B16] Ku K. H. Lee Y. J. Kim Y. Kim B. J. J. M. (2019). Shape-anisotropic diblock copolymer particles from evaporative emulsions: Experiment and theory. Macromolecules 52, 1150–1157. 10.1021/acs.macromol.8b02465

[B17] Lee J. Ku K. H. Park C. H. Lee Y. J. Yun H. Kim B. J. J. A. n. (2019). Shape and color switchable block copolymer particles by temperature and pH dual responses. Chem. Mater 13 (4), 4230–4237. 10.1021/acsnano.8b09276 30856312

[B18] Li X. Yi K. Ran Q. Fan Z. Liu C. Liu X. (2022). Selective removal of cationic organic dyes via electrospun nanofibrous membranes derived from polyarylene ethers containing pendent nitriles and sulfonates. Sep. Purif. Technol. 301, 121942. 10.1016/j.seppur.2022.121942

[B19] Liu Q. Chen J. Yang X. Qiao C. Li Z. Xu C. (2020). Synthesis, structure, and properties of N-2-hydroxylpropyl-3-trimethylammonium-O-carboxymethyl chitosan derivatives. Int. J. Biol. Macromol. 144, 568–577. 10.1016/j.ijbiomac.2019.12.125 31857162

[B20] Masoomi H. Wang Y. Chen C. Zhang J. Ge Y. Guo Q. (2021). A facile polymer mediated dye incorporation method for fluorescence encoded microbeads with large encoding capacities. Chem. Commun. (Camb) 57, 4548–4551. 10.1039/d0cc08202c 33956007

[B21] Pan H. Xu S. Ni Y. (2019). Rare-earth post-modified Zn-based coordination polymer microspheres: Simple room-temperature preparation, fluorescent performances and application for detection of tryptophane. Sens. Actuators B Chem. 283, 731–739. 10.1016/j.snb.2018.12.044

[B22] Pan J. Wang L. Shi Y. Li L. Xu Z. Sun H. (2022). Construction of nanodiamonds/UiO-66-NH_2_ heterojunction for boosted visible-light photocatalytic degradation of antibiotics. Sep. Purif. Technol. 284, 120270. 10.1016/j.seppur.2021.120270

[B23] Ran Q. Fan Z. Guo X. Li X. Yi K. Liu X. (2023). Simultaneous adsorption and fluorescent detection of Cr(VI) via lanthanide coordinating polymeric porous microparticles. Chem. Eng. J. 457, 141214. 10.1016/j.cej.2022.141214

[B24] Shin J. J. Kim E. J. Ku K. H. Lee Y. J. Hawker C. J. Kim B. J. (2020). 100th anniversary of macromolecular science viewpoint: Block copolymer particles: Tuning shape, interfaces, and morphology. ACS Macro Lett. 9, 306–317. 10.1021/acsmacrolett.0c00020 35648552

[B25] Steinhaus A. Chakroun R. Müllner M. Nghiem T.-L. Hildebrandt M. Gröschel A. H. (2019). Confinement assembly of ABC triblock terpolymers for the high-yield synthesis of Janus nanorings. ACS Nano 13, 6269–6278. 10.1021/acsnano.8b09546 31082201

[B26] Sun L. Xu H. Shao Y. Liu J. Fan L. J. (2019). Preparation and evaluation of fluorescent poly(p-phenyleneethylene) covalently coated microspheres with reactive sites for bioconjugation. J. Colloid Interface Sci. 540, 362–370. 10.1016/j.jcis.2019.01.009 30660793

[B27] Wang Q. Xie D. Chen J. Liu G. Yu M. (2021). Facile fabrication of luminescent rare-earth-doped PS/AA composites for anti-counterfeiting applications. J. Mater Sci. 56, 13146–13155. 10.1007/s10853-021-06133-4

[B28] Xu J. Wang K. Li J. Zhou H. Xie X. Zhu J. (2015). ABC triblock copolymer particles with tunable shape and internal structure through 3D confined assembly. Macromolecules 48, 2628–2636. 10.1021/acs.macromol.5b00335

[B29] Yan N. Liu X. Zhu J. Zhu Y. Jiang W. (2019). Well-ordered inorganic nanoparticle arrays directed by block copolymer nanosheets. ACS Nano 13, 6638–6646. 10.1021/acsnano.9b00940 31125524

[B30] Yang H. Hu L. Chen C. Zhao H. Wang P. Zhu T. (2020). Influence mechanism of fluorescent monomer on the performance of polymer microspheres. J. Mol. Liq. 308, 113081. 10.1016/j.molliq.2020.113081

[B31] You L. J. Song L. D. Huang C. Lu F. F. Xu K. Zhang Q. Q. (2019). Controllable preparation and high properties of fluorescence and surface enhanced Raman spectra encoded poly(glycidyl methacrylate) microsphere. Express Polym. Lett. 13, 37–51. 10.3144/expresspolymlett.2019.5

[B32] Zhan H. Chen J. Zhang C. Zhang J. Fan L.-J. (2021). Design, synthesis, and adhesion of fluorescent conjugated polymers with pendant catechol groups. ACS Appl. Polym. Mater 3, 4543–4553. 10.1021/acsapm.1c00603

